# Amnion and chorion matrix maintain hMSC osteogenic response and enhance immunomodulatory and angiogenic potential in a mineralized collagen scaffold

**DOI:** 10.3389/fbioe.2022.1034701

**Published:** 2022-11-14

**Authors:** Vasiliki Kolliopoulos, Marley J. Dewey, Maxwell Polanek, Hui Xu, Brendan A. C. Harley

**Affiliations:** ^1^ Chemical and Biomolecular Engineering, University of Illinois at Urbana-Champaign, IL, United States; ^2^ Department Materials Science and Engineering, University of Illinois at Urbana-Champaign, IL, United States; ^3^ Tumor Engineering and Phenotyping (TEP) Shared Resource, Cancer Center at Illinois, University of Illinois Urbana-Champaign, Urbana, IL, United States; ^4^ Carl R. Woese Institute for Genomic Biology, Urbana, IL, United States

**Keywords:** craniomaxillofacial defects, bone regeneration, amnion membrane, chorion membrane, collagen scaffold

## Abstract

Craniomaxillofacial (CMF) bone injuries present a major surgical challenge and cannot heal naturally due to their large size and complex topography. We are developing a mineralized collagen scaffold that mimics extracellular matrix (ECM) features of bone. These scaffolds induce *in vitro* human mesenchymal stem cell (hMSC) osteogenic differentiation and *in vivo* bone formation without the need for exogenous osteogenic supplements. Here, we seek to enhance pro-regenerative potential *via* inclusion of placental-derived products in the scaffold architecture. The amnion and chorion membranes are distinct components of the placenta that each have displayed anti-inflammatory, immunomodulatory, and osteogenic properties. While potentially a powerful modification to our mineralized collagen scaffolds, the route of inclusion (matrix-immobilized or soluble) is not well understood. Here we compare the effect of introducing amnion and chorion membrane matrix versus soluble extracts derived from these membranes into the collagen scaffolds on scaffold biophysical features and resultant hMSC osteogenic activity. While inclusion of amnion and chorion matrix into the scaffold microarchitecture during fabrication does not influence their porosity, it does influence compression properties. Incorporating soluble extracts from the amnion membrane into the scaffold post-fabrication induces the highest levels of hMSC metabolic activity and equivalent mineral deposition and elution of the osteoclast inhibitor osteoprotegerin (OPG) compared to the conventional mineralized collagen scaffolds. Mineralized collagen-amnion composite scaffolds elicited enhanced early stage osteogenic gene expression (BGLAP, BMP2), increased immunomodulatory gene expression (CCL2, HGF, and MCSF) and increased angiogenic gene expression (ANGPT1, VEGFA) in hMSCs. Mineralized collagen-chorion composite scaffolds promoted immunomodulatory gene expression in hMSCs (CCL2, HGF, and IL6) while unaffecting osteogenic gene expression. Together, these findings suggest that mineralized collagen scaffolds modified using matrix derived from amnion and chorion membranes represent a promising environment conducive to craniomaxillofacial bone repair.

## 1 Introduction

Craniomaxillofacial (CMF) bone defects can arise from congenital, post oncologic, or traumatic injuries such as cleft palate, tumor ablations, or traffic injuries respectively. Critical sized CMF bone defects are large and irregular in size such that they cannot heal naturally and instead require surgical intervention *via* grafts or other alternatives. In the US alone, approximately 500,000 bone graft procedures are performed annually (Greenwald et al., 2001). The gold standard to repair these injuries are autografts, which require bone to be excised from a secondary site from the patient which have limited availability and can lead to donor site morbidity. Alternatively, allografts, bone from a donor patient, are also widely used; however, these display decreased osteoinduction and osteogenic capability following sterilization (Greenwald et al., 2001).

Biomaterial and stem cell approaches are increasingly being developed to repair such bone injuries. The collagen and calcium phosphate mineral composition of bone have inspired a wide range of collagen biomaterials (Lynn et al., 2010; David et al., 2015; Quinlan et al., 2015; Sheehy et al., 2021). Stem cells can act as endogenous factories of bioactive molecules capable of recruiting host cells to the tissue site, modulating the local inflammatory microenvironment, and promoting angiogenesis (Caplan, 2007). Numerous studies have used biomaterials to sequester mesenchymal stem cell (MSC) produced secretome to modulate the behavior of other cell types such as immune and endothelial cells, highlighting the potential for using bioactive molecules contained by a biomaterial as indirect signals in the absence of direct MSC stimuli (Gionet-Gonzales et al., 2021; Wechsler et al., 2021). Our laboratory has developed a class of mineralized collagen-glycosaminoglycan scaffold that promotes MSC osteogenesis and mineral formation *in vitro* without the use of exogenous factors (e.g. BMP2) (Ren et al., 2015; Weisgerber et al., 2015). We also showed the secretome of MSCs within these scaffolds can be altered *via* inclusion of disparate glycosaminoglycans within the mineralized collagen scaffold matrix (Dewey et al., 2020a). However, the immune response after implantation can pose a barrier to bone repair, motivating efforts to develop strategies to boost pro-healing cell phenotypes in these scaffolds.

Placental derived membranes such as the amnion and chorion membrane display unique extracellular matrix and sequestered biomolecules including platelet derived growth factor BB (PDGF-BB), hepatocyte growth factor (HGF), tissue inhibitor of metalloproteinase 2 (TIMP-2), endocrine gland derived vascular endothelial growth factor (EG-VEGF) that hold promise for bone repair (Niknejad et al., 2008; Koob et al., 2015; Hortensius and Harley, 2016; Go et al., 2017; Lei et al., 2017; McQuilling et al., 2017; Tang et al., 2018; Dewey et al., 2020b; Sabouri et al., 2020). The amnion membrane has been adapted for bone regeneration applications. Dehydrated human amnion chorion membrane (dHACM) allografts can promote stem cell migration *in vitro*, stem cell recruitment into the wound site *in vivo*, and neovascularization (Maan et al., 2015a; Lei et al., 2017). Decellularized amnion membranes have been used as barriers *in vivo* to reduce fibroblast invasion, stabilize bone grafts, and aid bone growth (Li et al., 2015). Cryopreserved human amnion membrane reduced the inflammatory response of primary human macrophage to an inflammatory challenge (Witherel et al., 2017). The underlying mechanism by which the human amnion membrane demonstrates efficacy in promoting wound healing remains unknown. However, both amnion and chorion membrane extracts have been shown to contain a broad range of growth factors necessary for osteogenic differentiation and bone repair (Koob et al., 2015; Go et al., 2017; McQuilling et al., 2017; Go et al., 2021), suggesting a partial mechanism for the efficacy of human amnion membranes (Maan et al., 2015b). The chorion membrane contains more growth factors per cm^2^ compared to amnion (McQuilling et al., 2017) with soluble extracts derived from chorion promoting increased osteogenic differentiation and activity compared to amnion extracts (Go et al., 2017). Our lab has previously incorporated amnion membrane (AM) matrix into non-mineralized collagen scaffolds under development for tendon repair, showing increased progenitor cell metabolic activity in response to an inflammatory challenge (Hortensius et al., 2016; Hortensius and Harley, 2016). While rapid degradation and clearance of AM matrix may limit its practical application, inclusion within a calcium and phosphate functionalized scaffold may reduce this degradation (Sabouri et al., 2020). We showed direct incorporation of AM matrix into mineralized collagen scaffolds improved MSC osteogenesis and mineral formation in response to a soluble inflammatory challenge (Dewey et al., 2020b). However, there remains a need to understand whether inclusion of membrane matrix *vs*. soluble isolates from the amnion *vs*. chorion membrane are a route to improve processes (osteogenesis, matrix deposition, angiogenesis) associated with bone regeneration in mineralized collagen scaffolds.

In this article, we examine whether the amnion or chorion regenerative potential is driven by matrix bound or soluble isolates when deployed within a mineralized collagen scaffold. We define the effect of membrane matrix inclusion on scaffold pore size, mechanics, and factor release kinetics. We subsequently examine the decoupled influence of the matrix incorporated versus soluble extract components on *in vitro* osteogenic activity of mesenchymal stem cells. Together, this works provides a pathway for expanded use of amnion and chorion derived matrix and soluble products for future regenerative purposes.

## 2 Materials and methods

### 2.1 Experimental design

**FIGURE 1:**
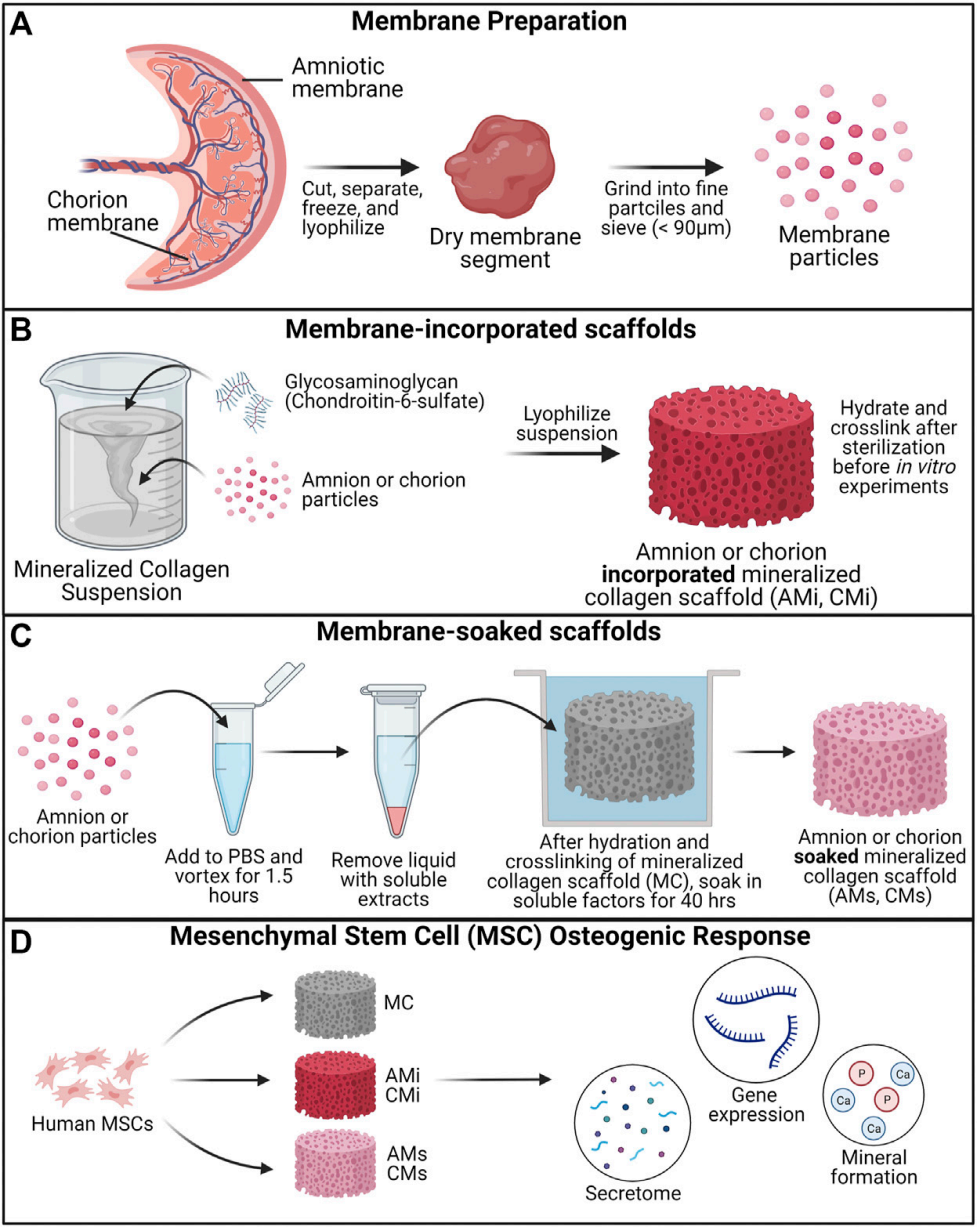
Experimental Outline. **(A)** Placental derived amnion and chorion membranes were harvested, decellularized and pulverized into fine particles (<90 μm diameter). Mineralized collagen scaffold variants were fabricated into: amnion or chorion incorporated or soaked. **(B)** Mineralized collagen—amnion or chorion incorporated scaffolds were fabricated by adding amnion or chorion particles during the scaffold fabrication. **(C)** To fabricate the soaked variants mineralized collagen scaffolds soaked in a media suspension containing amnion or chorion extracts. **(D)** Human mesenchymal stem cells were seeded on scaffolds and allowed to culture for 21 days. hMSC osteogenesis and immunomodulatory potential was determined through the evaluation of the genomic and proteomic expression and degree of mineral deposition.

The goal of this study was to determine the influence of the incorporation of pulverized placental derived human amnion and chorion membranes or their extracts within mineralized collagen scaffolds on hMSC osteogenesis. Pulverized amnion or chorion membrane particles were incorporated into mineralized collagen-glycosaminoglycan scaffolds during the scaffold fabrication process (AMi, CMi); alternatively, mineralized collagen scaffolds were soaked in soluble amnion or chorion extracts post-fabrication (AMs, CMs). Unconfined compression and porosimetry analyses were used to identify the effects of incorporation of the amnion or chorion matrix particles on scaffold mechanics. Furthermore, amnion and chorion extracts were screened with a cytokine array to identify their protein content. hMSCs were subsequently seeded onto amnion or chorion incorporated, amnion or chorion soaked, or (control) mineralized collagen scaffolds (MC). Cell viability, alkaline phosphate activity, mineral content, osteogenic gene and protein expression were evaluated over 21 days (Figure 1).

### 2.2 Amnion and chorion processing

A de-identified human placenta was obtained from Carle Medical Hospital (Urbana, IL) using an established materials transfer agreement. Use of de-identified placental matrix for this study was determined by the University of Illinois Office for the Protection of Research Subjects to not meet the definition of human subjects research and did not require Institutional Review Board approval for their use. Placental matrix is processed within 6 h of childbirth in a sterile biosafety hood. The amnion membrane was separated from the chorion membrane with tweezers and the umbilical cord was cut with sterile scissors. Amnion and chorion membranes were cut into smaller pieces and washed in PBS and finally in water before snap-freezing in liquid nitrogen and storing at −80°C. In a subsequent step, the amnion and chorion were lyophilized twice, first starting at 20°C, cooling to −40°C at a rate of 1°C/min, holding the frozen matrix at −40°C for 2 h, then sublimating the frozen matrix at a pressure of 0.2 Torr and a temperature of 0°C, to evaporate ice crystals (Hortensius et al., 2018; Dewey et al., 2020b). Cellular debris was then removed by soaking in a PBS solution containing 125 μg/ml thermolysin (Thermolysin from *Geobacillus stearothermophilus*, Sigma, MO, United States) for 20 min, with the treated amnion or chorion membranes washed in PBS then lyophilized for a second time before storing dry at −80°C (Hortensius et al., 2016).

### 2.3 Extracting and characterizing AM and CM factors from membranes

**FIGURE 2:**
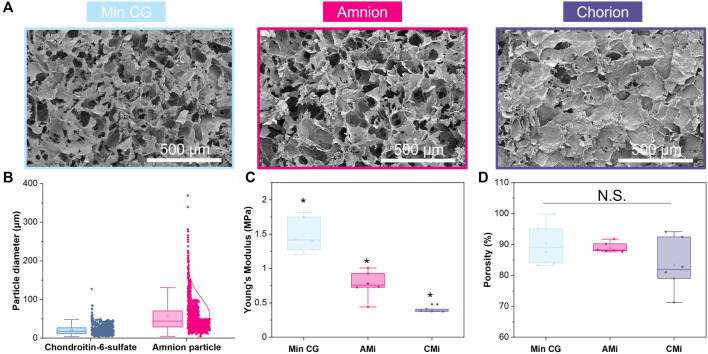
Characterization of mineralized collagen (MC), amnion or chorion incorporated membrane (AMi, CMi, and respectively) particle scaffolds. **(A)** Scanning electron microscopy (SEM) images of mineralized collagen, mineralized collagen-amnion, and mineralized collagen-chorion scaffolds. **(B)** Pulverized amnion and chorion particle size compared to the particle size of the glycosaminoglycan in the scaffolds. **(C)** Compression testing of mineralized collagen scaffold variants using an Instron mechanical tester (*n* = 6). * indicates all groups are significant from one another (*p* < 0.05). **(D)** Porosity as measured by wet-dry weights using IPA.

Lyophilized amnion and chorion were added to metal tubes with four 3.2 mm stainless steel beads, then pulverized in a Mini Beadbeater-24 (Biospec Products, OK) (5 cycles, 30 sec with intermittent flash freezing in liquid nitrogen for 10 sec). Pulverized matrix was screened using a 90 µm sieve (Hogentogler & Co Inc, MD) and particle size was compared to that of glycosaminoglycan (Figure 2B). 20 mg of amnion and chorion powder were added to 1.5 ml centrifuge tubes (separately) with 1 ml of PBS (20 mg/ml) then vortexed for 1 h to collect soluble factors. After vortexing, tubes were centrifuged and PBS was collected and stored at −80C until use in a cytokine array. A RayBiotech Cytokine array (AAH-CYT-5, lot 1207207043) was used to quantify protein release from the amnion and chorion. Three replicates were used for each membrane and 1 ml of the eluted factors in PBS was added to each membrane for 2 h with clean PBS used as a control (Supplementary Tables S1–S3).

### 2.4 Mineralized collagen-glycosaminoglycan scaffold fabrication

Mineralized collagen-glycosaminoglycan scaffolds were fabricated *via* lyophilization from mineralized collagen precursor suspensions as previously described (Harley et al., 2010; Weisgerber et al., 2015; Dewey et al., 2020a). Briefly, type I bovine collagen (1.9 w/v% Sigma-Aldrich, Missouri United States), calcium salts (0.9 w/v% calcium hydroxide and 0.4 w/v% calcium nitrate tetrahydrate, Sigma-Aldrich), and chondroitin-6-sulfate glycosaminoglycan (0.84 w/v%, CS6, Chondroitin sulfate sodium salt from shark cartilage, CAS Number 9082-07-9, Sigma-Aldrich) were homogenized in mineral buffer solution (0.1456 M phosphoric acid/0.037 M calcium hydroxide). Amnion and chorion incorporated scaffold variants (AMi and CMi, respectively) were fabricated by adding 0.58 g of amnion or chorion powder (particle size <90 μm) into 150 ml of slurry (3.8 mg/ml) during homogenization resulting in a 5:1 w:w ratio of collagen:amnion or chorion due to the high collagen content in the amnion and chorion membranes. Scaffold precursor suspensions (MC, AMi, and CMi) were pipetted into alumnum molds and lyophilized using a Genesis freeze-dryer (VirTis, Gardener, New York United States). Suspensions were cooled at a constant rate of 1 ⁰C/min from 20°C to -10°C followed by a hold at to −10°C for 2 h. The frozen suspension was subsequently sublimated at 0°C and 0.2 Torr, resulting in a porous scaffold network. After lyophilization, a 6 mm diameter biopsy punch (Integra LifeSciences, New Jersey, United States) was used to create individual scaffolds.

Amnion and chorion soaked scaffold variants (AMs and CMs, respectively) were fabricated by taking lyophilized and sterilized (see section 2.7) 6 mm mineralized collagen scaffold specimens and soaking them during the final step of hydration in phenol-free media containing 3.8 mg/ml of amnion or chorion extracts (described in section 2.3), corresponding to the concentration of amnion and chorion particles in the incorporated variants, for 48 h prior to cell seeding (see section 2.8 for details on sterilization and hydration).

### 2.5 Scanning electron microscopy of mineralized collagen, collagen-amnion, and collagen-chorion scaffolds

Scanning electron microscopy (SEM) was used to visualize the macroscopic pore architecture of the conventional mineralized collagen scaffolds (MC) as well as amnion and chorion incorporated (AMi, and CMi) scaffolds. Dry non-crosslinked scaffolds were cut to expose the interior of the scaffolds before sputter coating with Au/Pd (Denton Desk II TSC, New Jersey, United States). After sputter-coating, samples were imaged using an FEI Quanta FEG 450 ESEM (FEI, Hillsboro, OR) under high vacuum (Figure 2A).

### 2.6 Unconfined compression testing of mineralized collagen, collagen-amnion, and collagen-chorion scaffolds

The mechanical behavior of conventional mineralized collagen scaffolds (MC) as well as amnion and chorion incorporated (AMi, and CMi) scaffolds was quantified using an Instron 5943 mechanical tester (Instron, Norwood, MA, United States) with a 100 N load cell (dry conditions; 12 mm diameter acellular scaffold disks) as previously described (*n* = 6, Figure 2C) (Dewey et al., 2021a). Briefly, MC, AMi, and CMi scaffolds were compressed to failure (2 mm/min), with the resultant stress-strain curves used to determine the Young’s modulus using analysis techniques appropriate for low-density open-cell foams (Harley et al., 2007; Kanungo et al., 2008; Kanungo and Gibson, 2010). Mechanical properties of dry scaffold specimens are reported, and previous efforts have described the global effect of scaffold hydration and 1-Ethyl-3-(3-dimethylaminopropyl)carbodiimide crosslinking on mineralized collagen scaffolds (11-fold reduction in Young’s modulus and compressive stress) (Harley et al., 2007; Kanungo et al., 2008; Qi Zhou et al., 2021).

### 2.7 Porosity measurements of mineralized collagen, collagen-amnion, and collagen-chorion scaffolds

The porosity of mineralized collagen, amnion and chorion incorporated (MC, AMi, and CMi) scaffolds was evaluated using a previously described solvent exchange method (*n* = 6, Figure 2D) (Ajaxon et al., 2015). The apparent volume (the volume described by the outer dimensions of the specimen) and dry weight of each scaffold was measured. Scaffolds were then soaked in isopropanol for 24 hrs and weighed to record the wet weight. The volume of the pores was calculated from the difference between the wet and dry weight accounting for the density of isopropanol.

### 2.8 Sterilization, hydration, crosslinking, and creation of amnion/chorion soaked scaffolds

All scaffolds were placed in sterilization pouches and sterilized *via* ethylene oxide treatment for 12 hrs using a AN74i Anprolene gas sterilizer (Andersen Sterilizers Inc., Haw River, NC, United States). After sterilization, all subsequent steps proceeded using aseptic techniques. Sterile scaffolds were then hydrated and crosslinked using previously described EDC-NHS chemistry (Caliari and Harley, 2011; Caliari and Harley, 2014; Dewey et al., 2020a; Tiffany et al., 2020; Dewey et al., 2021b). Briefly, scaffolds were soaked in 100% ethanol, then washed multiple times in phosphate buffered saline (PBS), followed by EDC-NHS crosslinking. Conventional mineralized (MC), AMi, and CMi scaffolds were then washed in PBS then soaked in normal growth media (low glucose Dulbecco’s Modified Eagle Medium, 10% mesenchymal stem cell fetal bovine serum (Gemini, CA, United States), and 1% antibiotic-antimycotic (Gibco, MA, United States)) for 48 h prior to cell seeding. Alternatively, to fabricate the AMs and CMs variants, MC scaffolds were soaked for 48 h in cell culture media containing either amnion extracts or chorion extracts (3.8 mg/ml) prior to cell seeding.

### 2.9 Quantifying biomolecule release from acellular amnion and chorion scaffolds

The release of biomolecules from amnion/chorion incorporated *vs*. amnion/chorion soaked scaffolds was determined after incubation of scaffolds in 1 ml of PBS on a shaker in an incubator (37°C and 5% CO_2_) for 21 days. PBS was exchanged every 3 days and stored at −20°C until further use. An OPG ELISA (R&D Systems) was used with 100 μl of sample with PBS as a background control. Cumulative release curves of OPG from scaffolds were used to evaluate the potential release of growth factors and biomolecules from amnion- and chorion-containing scaffolds (*n* = 6) (Supplementary Figure S1)

### 2.10 Mesenchymal stem cell culture on scaffolds

Human bone-marrow derived mesenchymal stem cells (hMSCs, female, age 20, RoosterBio, MD, United States) were expanded at 37°C and 5% CO_2_ in RoosterNourish™-MSC expansion medium (RoosterBio) until passage 5. During culture, cells were routinely tested for *Mycoplasma* with a MycoAlert™ *Mycoplasma* Detection Kit (Lonza, Switzerland). hMSCs were seeded on scaffolds in Costar ^®^ ultra-low attachment plates (Corning, NY, United States) *via* a previously described static seeding method: 50,000 cells in 10 µl media were pipetted into one surface of the scaffold then allowed to rest in an incubator (37°C and 5% CO_2_) for 30 min; scaffolds were subsequently flipped over and another 50,000 cells in 10 µl media added before leaving scaffolds in the incubator for 1.5 h to facilitate cell attachment (total 100,000 hMSCs per scaffold). After this, 1 ml of complete mesenchymal stem cell growth media (low glucose Dulbecco’s Modified Eagle Medium, 10% mesenchymal stem cell fetal bovine serum (Gemini, CA, and United States), and 1% antibiotic-antimycotic (Gibco, MA, and United States)) without osteogenic supplements was added to each well. Phenol-red free complete mesenchymal stem cell medium was used for ELISA and alkaline phosphatase activity samples as phenol-red interferes with the absorbance readings of these assays. Cell-seeded scaffolds were maintained in an incubator (37°C and 5% CO_2_) with medium replacements every 3 days for up to 21 days.

### 2.11 Cell viability quantification

**FIGURE 3:**
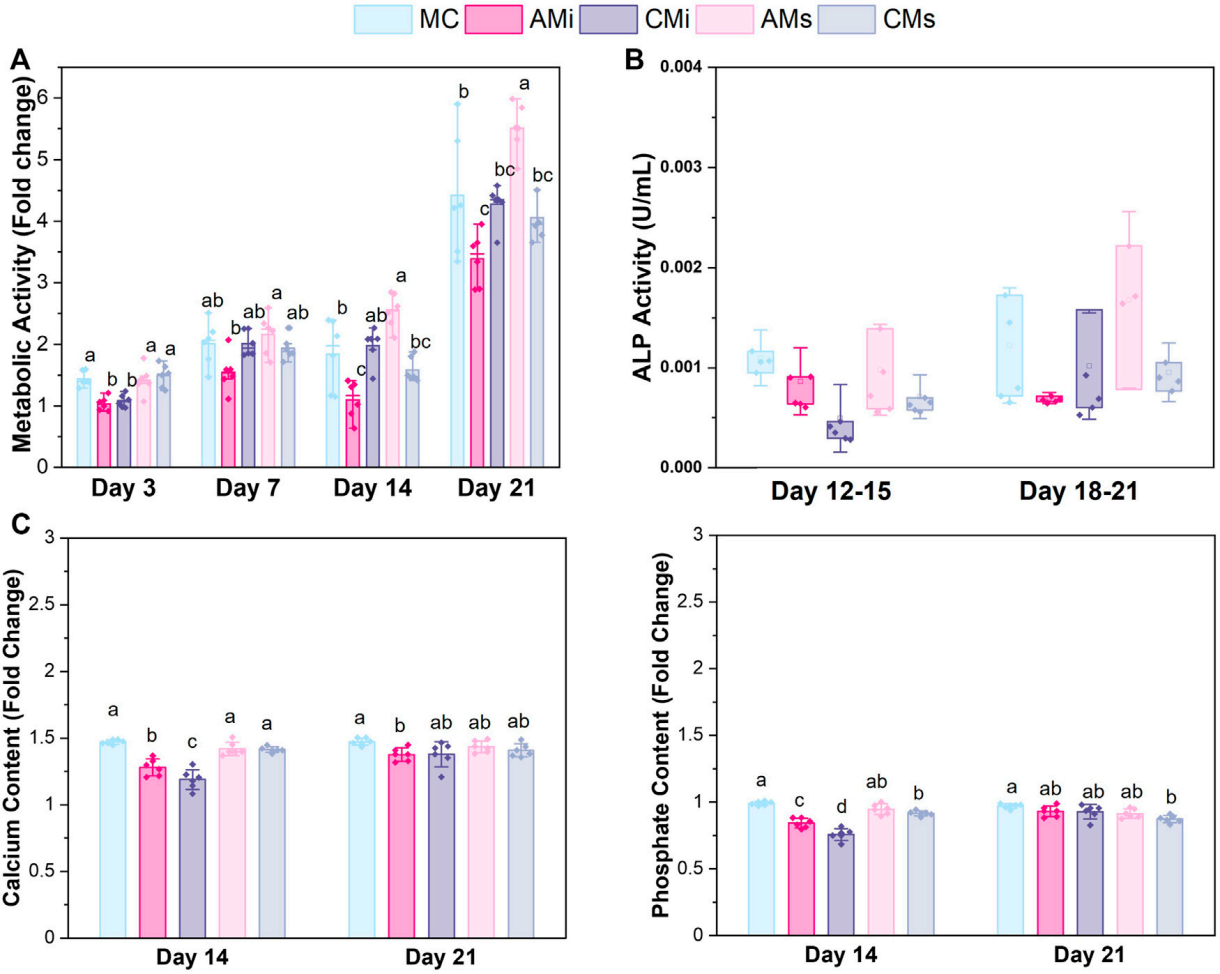
hMSCs cultured on scaffold variants for 21 days. **(A)** Metabolic activity measured by a non-destructive alamarBlue^®^ assay over the course of 21 days expressed as a fold change to a day 0 control (*n* = 6). Different letter indicated significance at a level of *p* < 0.05 within the same day. **(B)** Cell dependent bone formation measured by alkaline phosphate (ALP) activity assay pooled from scaffolds across 15 and 21 days (*n* = 6). **(C)** Amounts of calcium and phosphate produced by hMSCs on days 14 and 21 measured by inductively coupled plasma (ICP) mass spectroscopy (*n* = 6). Different letter indicated significance at a level of *p* < 0.05 within the same day.

Metabolic activity of hMSC seeded scaffolds was determined using a non-destructive alamarBlue^®^ assay (Invitrogen, Carlsbad, CA, and United States) at days 3, 7, 14, and 21 (*n* = 6) (Figure 3A). Scaffolds were rinsed in PBS prior to incubation in alamarBlue^®^ under gentle shaking in an incubator at 37°C for 2 h. Following incubation, the alamarBlue^®^ solution was measured for the fluorescence of resorufin (540 (52)-nm excitation, 580 (20-nm emission) using a F200 spectrophotometer (Tecan, Mannedorf, and Switzerland). Metabolic activity was calculated from a standard curve generated on Day 0 from a known number of cells, with results normalized to the initial cell seeding density of 100,000 cells.

### 2.12 Alkaline phosphate activity

An alkaline phosphatase (ALP; Abcam, England) activity assay was used to determine cell-dependent osteogenic activity (*n* = 6) (Figure 3B). Results were compared between media isolated from cell-seeded scaffold groups between 15 and 21 days of culture, using Phenol-red free complete mesenchymal stem cell medium as a background control. P-nitrophenyl phosphate (pNPP, µmol) concentration per well was converted to U/well with known reaction time and volume of sample.

### 2.13 Quantifying calcium and phosphorous mineral deposition

Inductively coupled plasma (ICP) mass spectroscopy was used to assess the amount of calcium and phosphorous produced by hMSCs seeded on scaffold variants (Figure 3C). Briefly, scaffolds were added to Formal-Fixx (10% neutral buffered formalin, ThermoFisher Scientific) for 24 h at 4°C, washed in PBS three times for 5 min each, partially dried *via* blotting (Kimwipe), stored at -80°C, then dried *via* lyophilization prior to analysis. ICP optical emission spectrometry (OES) was performed on fixed, dried scaffolds (*n* = 6 per group). Samples were weighed prior to being dissolved in concentrated nitric acid (Trace Metal Grade concentrated HNO_3_, Thermo Fischer Scientific 67%–70%) then subjected to automated sequential microwave digestion (CEM Mars 6 microwave digester). The resulting acidic solution was diluted to a volume of 50 ml using DI water to a final concentration <5% acid. ICP-OES was calibrated with a series of matrix matched standards before introducing unknown collagen samples. Digestion and ICP-OES analysis parameters are listed in Supplementary Table S4 **.**

### 2.14 OPG and OPN released factors from cell-seeded scaffolds

**FIGURE 4:**
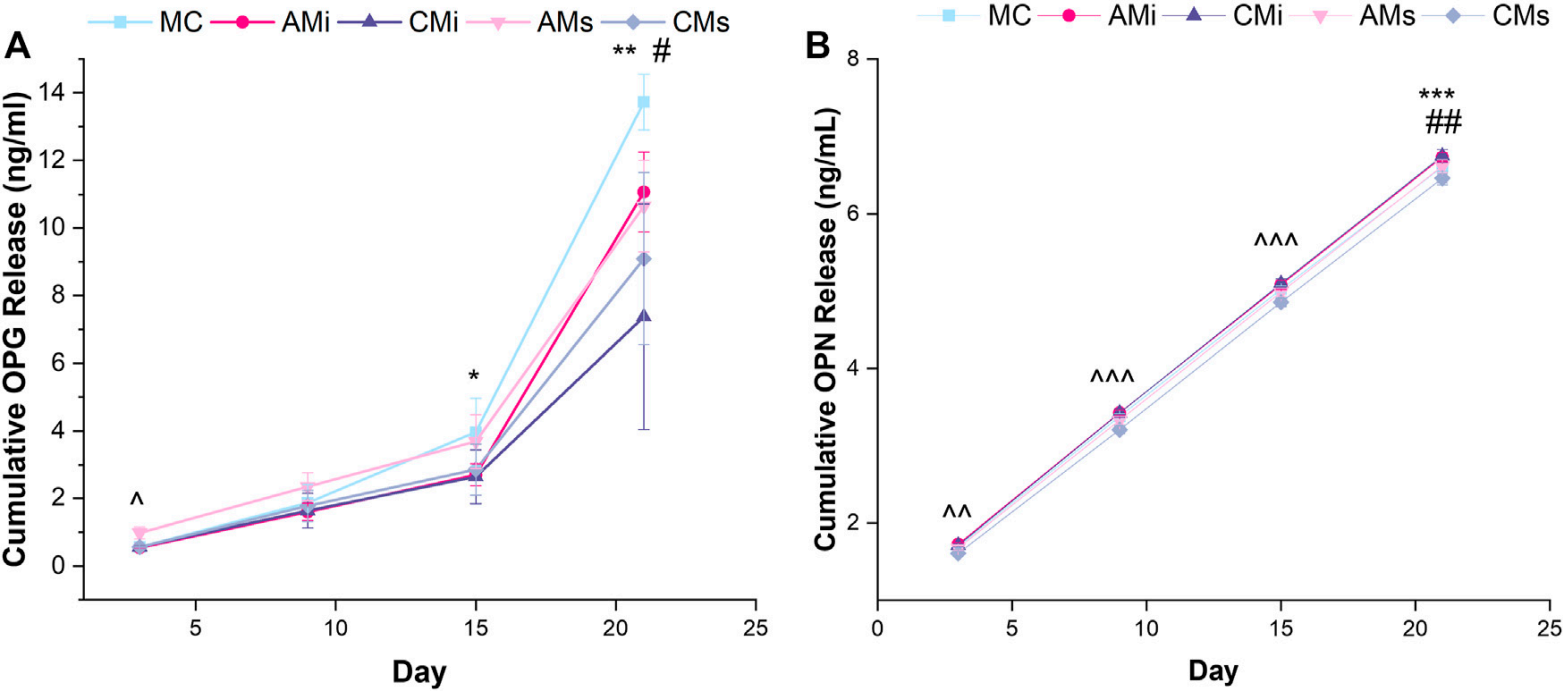
Osteoprotegerin (OPG) and osteopontin (OPN) cumulative release from hMSC-seeded mineralized collagen scaffolds (MC) containing amnion or chorion particles (AMi or CMi) or amnion or chorion extracts (AMs or CMs) measured using ELISAs. **(A)** Mesenchymal stem cells produce OPG to inhibit osteoclastogenesis.* denotes that MC has significantly (*p* < 0.05) greater release of OPG than CMi, ** denotes that MC has significantly (*p* < 0.05) greater release of OPG than both chorion scaffold variants, ^ denotes that AMs is significantly (*p* < 0.05) greater than all other groups, and # denotes that AMi releases significantly greater OPG than CMs. **(B)** Osteopontin (OPN) is an indication of mesenchymal stem cell osteogenic differentiation. ^^ denotes that AMs, CMs, and AMi release significantly (*p* < 0.05) different levels of OPN, ^^^ denotes that the soaked scaffolds (AMs, CMs) release significantly (*p* < 0.05) different levels of OPN compared to the incorporated scaffolds (AMi, CMi), ## denotes that MC has significantly (*p* < 0.05) greater release of OPN compared to AMi and CMi, and *** denotes that CMi releases significantly (*p* < 0.05) different levels of OPN than AMs. Data represented as average ±standard deviation (*n* = 6).

The amount of osteoprotegerin (OPG) and osteopontin (OPN) released for hMSC-seeded scaffolds was quantified *via* an OPG (DY805, R&D Systems, Minnesota, United States) and OPN (DY1433, R&D Systems, Minnesota, United States) ELISA respectively (Figure 4). Media was collected every 3 days throughout the 21 days culture period then pooled (Day 3; Day 6 and 9; Day 12 and 15; Day 18 and 21) for analysis, comparing result to a blank media control (*n* = 6).

### 2.15 RNA isolation and gene expression analysis from cell-seeded scaffolds

Scaffolds seeded with hMSCs were harvested for RNA isolation on days 3, 7, 14, and 21. Each hMSC seeded scaffold was placed in a 2 ml reinforced microvial (BioSpec Products, Oklahoma, United States) containing four 3.2 mm stainless steel beads and 1 ml of TRIzol™ Reagent (ThermoFisher Scientific, Massachusetts, and United States). A conventional TRIzol isolation protocol was followed using the RNEasy mini kit (Qiagen) (Witherel et al., 2018). Briefly, scaffolds underwent seven pulverization cycles in 1 ml of TRIzol for 15 s intervals followed by 20 s rest on ice until fully homogenized. Chloroform (200 ul) was then added to each tube and allowed to rest for 3 min. The tubes were then centrifuged at 15,000 rpm for 15 min at 4°C to separate the RNA, DNA, and organic layers. Isolated RNA was then processed through the RNEasy mini kit per the manufacturer’s instructions with RNA concentration measured using a NanoDrop spectrophotometer.

**FIGURE 5:**
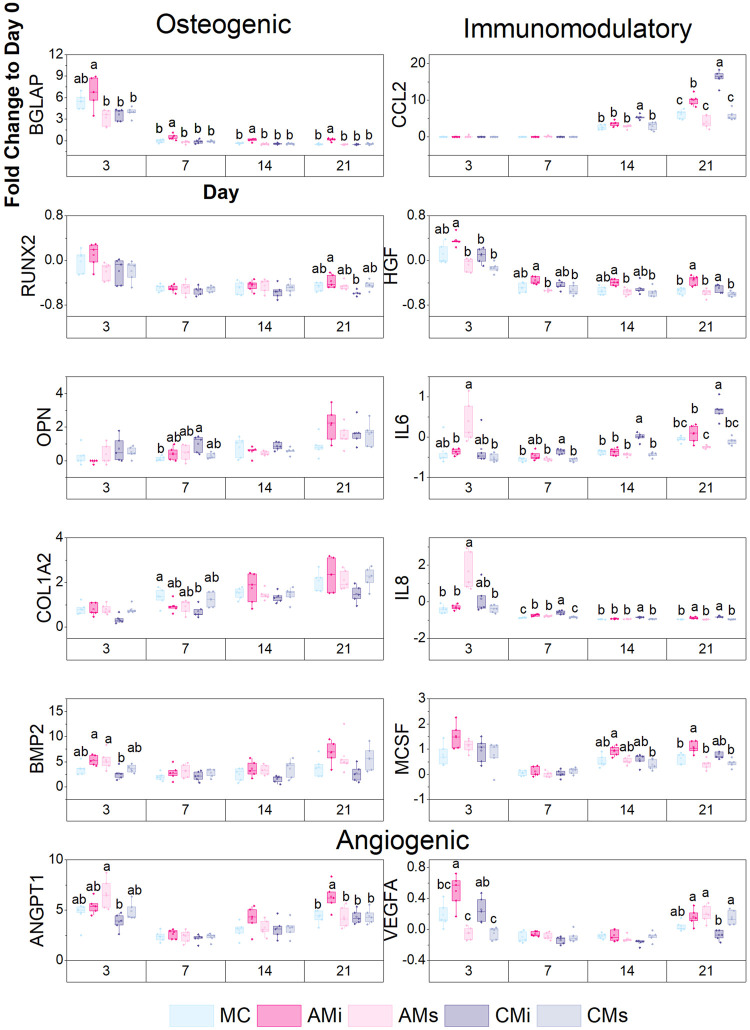
A custom NanoString code set was used to measure hMSC osteogenic and immunomodulatory gene expression in response to scaffold content (mineralized collagen, MC; mineralized collagen amnion incorporated, AMi; mineralized collagen chorion incorporated, CMi; mineralized collagen amnion extracts, AMs; mineralized collagen chorion extracts, CMs). Gene expression is represented as a fold change compared to hMSC gene expression prior to seeding on the scaffolds, normalized to GAPDH (*n* = 5). Different letter indicated significance at a level of *p* < 0.05 within the same day.

Transcript expression was also quantified with the NanoString nCounter System (NanoString Technologies, Inc.) using a custom panel of 38 mRNA probes Supplementary Table S5. The NanoString nCounter System identifies and counts individual transcripts without requiring reverse transcription or amplification through the use of unique color-coded probes. Isolated RNA was quantified using Qubit RNA BR Assay Kit and loaded to cartridges to run the NanoString assay as instructed by the manufacturer. The nSolver Analysis Software (NanoString Technologies, Inc.) was used for data processing, normalization, and evaluation of expression. Raw data was normalized to three housekeeping genes (GAPDH, GUSB, and OAZ1) and day 0 controls (*n* = 5). Expression levels are depicted as a fold change (Figure 5).

### 2.16 Statistics

Statistics were performed using OriginPro (OriginPro, Massachusetts, United States) and RStudio (RStudio, Massachusetts, and United States) software. Significance was set to *p* < 0.05. First, a Shapiro-Wilk test was used to test for normality, followed by a Grubbs test to remove outliers if data was not normal. If removal of outliers did not result in normal data, the outlier was not removed from the data set. A Levene’s test was also performed to test the equal variance assumption. For normal data with equal variance, an ANOVA with a Tukey post-hoc test was used to assess significance. If data was normal but had unequal variance, a one-way Welch’s ANOVA and Welch/Games-Howell post-hoc was performed to determine significance. In the case of non-normal data with unequal variance, a Welch’s Heteroscedastic F test and Welch/Games-Howell post-hoc were performed. Finally, if data was non-normal but had equal variance, a Kruskal-Wallis test was used. Error bars for all data are represented as mean ± standard deviation, and all graphs were made in OriginPro.

## 3 Results

### 3.1 Addition of the amnion and chorion membrane matrix influences scaffold mechanical properties but not porosity

The particle size of amnion membrane derived matrix added to the scaffolds showed a similar size distribution as the chondroitin 6-sulfate glycosaminoglycan also used to fabricate mineralized collagen scaffolds (Figure 2B). The amnion and chorion membrane containing mineralized collagen scaffolds (AMi, CMi) displayed qualitatively similar open-pore microstructures to conventional mineralized collagen scaffolds visible by SEM imaging (MC) (Figure 2A). The conventional MC scaffold displayed a significantly greater Young’s Modulus than AMi or CMi scaffold variants (*p* < 0.05), with CMi scaffolds displaying a significantly reduced Young’s Modulus compared to AMi variants (*p* < 0.05) (Figure 2C). Addition of amnion or chorion membrane matrix did not significantly influence the macroscopic percent porosity of the scaffolds (Figure 2D).

### 3.2 Cytokine analysis of amnion and chorion derived matrix reveal pro-regenerative factors

Analysis of the cytokines released by the amnion and chorion membranes revealed significant release of osteogenic, osteoclastogenic, pro-inflammatory, anti-inflammatory, and angiogenic factors (Supplementary Tables S1–S3). Amnion and chorion matrix both release high levels of: IGFBP-1, a potent regulator of bone mass and osteoblast differentiation; OPN, an osteogenic protein that plays key roles in late-stage bone formation and mineralization (Bailey et al., 2017); TIMP-2, an anti-inflammatory protein and inhibitor of metalloproteinases (MMPs); and Angiogenin, a regulator of neovascularization including endothelial migration, proliferation, and differentiation. Amnion matrix released significantly greater (*vs*. Chorion): OPG (*p* < 0.0001), a known osteoclastogenesis inhibitor; and IL-8 (*p* < 0.001), a regulator of osteoclastogenesis, bone-resorption, neutrophil recruitment, and angiogenesis. Chorion matrix released significantly greater (*vs*. Amnion): Leptin (*p* < 0.05), a regulator of bone growth and metabolism (Upadhyay et al., 2015); RANTES (*p* < 0.05), a regulator of leukocyte migration, angiogenesis, and wound healing; and HGF (*p* < 0.01), a stimulator of osteoclast resorptive capacity.

We previously showed mineralized collagen scaffolds can sequester 60%–90% of growth factors from solution (Tiffany et al., 2020). As OPG plays a vital role in osteoclastogenesis inhibition and was found to be released in high levels from both membranes (Supplementary Table S1). We subsequently examined OPG release profiles of incorporated and soaked scaffolds comparing results to the conventional mineralized collagen scaffold (MC). Similar OPG release profiles were observed over 21 days for all groups suggesting that significant fractions of growth factors contained within CM and AM matrix are being retained in the scaffold (Supplementary Figure S1).

### 3.3 Incorporating soluble amnion extracts in mineralized collagen scaffolds increases MSC metabolic activity

The metabolic activity of the hMSCs seeded on all scaffold variants were traced over the course of 21 days. The metabolic activity of hMSCs in amnion extract-soaked scaffolds (AMs) was significantly greater than the amnion incorporated variants (AMi) across all days (*p* < 0.05) (Figure 3A). hMSCs in AMs scaffolds displayed significantly greater metabolic activity compared to conventional mineralized collagen scaffolds at days 14 and 21. hMSCs in AMs scaffolds also showed significantly higher metabolic activity than hMSCs in scaffolds containing Chorion membrane soluble extracts (CMs) on days 14 and 21 and scaffolds containing Chorion matrix (CMi) at day 21 (*p* < 0.05). Interestingly, hMSCs in scaffolds containing amnion matrix (AMi) showed significantly lower metabolic activity compared to the mineralized collagen control throughout the 21 day period. Lastly, incorporation of chorion signals either in matrix (CMi) or soluble (CMs) form did not influence hMSC metabolic activity compared to the mineralized collagen scaffold on days 7 through 21.

### 3.4 Soaking soluble amnion extracts in mineralized collagen scaffolds maintained mineral formation in scaffolds

Functional changes in mineral formation in hMSC seeded scaffolds were assessed *via* ICP and ALP assays. No significant (*p* < 0.05) differences in ALP activity, a proxy for cell-dependent mineral formation, were found between scaffold groups over the 21 days experiment (Figure 3B). Examining changes in calcium and phosphorous mineral content *via* ICP, we observed increased calcium and phosphorous in mineralized collagen scaffolds functionalized with amnion or chorion soluble extracts (AMs and CMs) compared to the incorporated groups (Figure 3B). However, scaffolds that had amnion or chorion matrix (AMi and CMi) displayed reduced calcium and phosphorous mineral across all timepoints, while scaffolds soaked in Chorion derived soluble extracts (CMs) showed reduced calcium and phosphorous mineral content compared to the mineralized scaffold control at the experimental endpoint (21 days; *p* < 0.05; Figure 3B).

### 3.5 Mineralized collagen scaffolds containing amnion or chorion matrix display reduced secretion of OPG and OPN compared to the conventional mineralized collagen scaffold

We evaluated endogenous production of osteogenic factors OPG and OPN from hMSCs as a function of inclusion of amnion and chorion matrix or soluble extract functionalization in mineralized collagen scaffolds for up to 21 days. OPG secretion was significantly increased at day 3 for hMSCs in mineralized collagen scaffolds containing amnion soluble extracts compared to all other groups (AMs; *p* < 0.05). At day 15 conventional mineralized collagen (MC) scaffolds expressed significantly more OPG than the chorion incorporated variant (CMi) (*p* < 0.05). By day 21, OPG production was highest in conventional mineralized collagen (MC) then scaffolds containing chorion matrix or soluble extracts (CMi, CMs) (Figure 4A). OPN secretion also increased for all groups over time (Figure 4A). Notably, hMSCs in mineralized collagen scaffolds containing soluble extracts derived from amnion or chorion membrane (AMs and CMs) showed significantly (*p* < 0.05) increased OPN secretion compared to versions including incorporated matrix (AMi, CMi) across 21 days of culture (Figure 4B). OPN secretion in amnion or chorion soaked scaffolds was not significantly (*p* < 0.05) different from the conventional mineralized collagen scaffold control.

### 3.6 NanoString analysis reveals a potential immunomodulatory and angiogenic role for amnion and chorion membrane matrix directly incorporated into the mineralized collagen scaffold

Lastly, we examined shifts in hMSC osteogenic, immunomodulatory, and angiogenic gene expression patterns for a library of genes in a custom NanoString panel as a function scaffold content. Scaffolds containing amnion matrix (AMi) displayed significantly higher BGLAP (bone remodeling) expression (*p* < 0.05) compared to all other groups by day 21 (Figure 5). Scaffolds containing chorion matrix displayed enhanced OPN expression and decreased COL1A2 expression compared to the conventional mineralized collagen (MC) at day 7. Overall, all groups showed an increase in OPN and COL1A2 expression over the 21 days. Both amnion groups displayed significantly higher expression of BMP2 compared to the chorion incorporated variant at day 3. We also examined immunomodulatory genes including CCL2 (monocyte chemotaxis), IL-6 (acute inflammation), IL-8 (chronic inflammation), and HGF (MSC-secreted immunomodulatory protein) (Caplan, 2007; Weiss and Dahlke, 2019; Weiss and Hendrik Dahlke, 2019; Wong et al., 2020e; Katagiri et al., 2021). Scaffolds containing amnion and chorion matrix displayed significantly upregulated expression of CCL2 and IL-6 (*p* < 0.05) compared to the conventional mineralized scaffold and those soaked in amnion or chorion derived soluble factors at day 21. CCL2 and IL-6 expression also increased with time (day 14–21) in scaffolds containing amnion or chorion matrix. Scaffolds containing amnion and chorion matrix also displayed significantly higher expression of IL-8 and HGF (*p* < 0.05) compared to all other groups at day 21. Further, scaffolds containing amnion matrix also displayed a significant upregulation of angiogenic genes (VEGFA, ANGPT1), with ANGT1 significantly upregulated in amnion incorporated scaffolds (AMi) compared to all others at day 21, while VEGFA was significantly upregulated in amnion incorporated scaffolds at day 3.

## 4 Discussion

Biomaterials were once designed to be inert and avoid the induction of the host inflammatory response. In recent years, the complex cell interactions at the wound site are increasingly believed to be a powerful tool to be leveraged to improve repair. There is a significant opportunity to develop biomaterials for CMF defects that promote osteogenesis and temporally modulate the inflammatory response to accelerate healing. In this study, we describe the fabrication of a series of mineralized collagen scaffold variants to include matrix or soluble biomolecules isolated from human amnion or chorion membrane. Previous work by Starecki et al. (2014) showed amnion membrane containing scaffolds significantly enhanced bone formation in critical size femoral defects in adult Sprague-Dawley rats (Starecki et al., 2014). Similarly, Akhlaghi et al. (2019) showed human amnion membrane could improve maxillomandibular bone repair; however, the source of these regenerative properties was not well understood (Akhlaghi et al., 2019). Prior work suggested that the immunomodulatory and pro-regenerative potential of placental derived membranes (Amnion, Chorion) may lie in their matrix or entrapped soluble factor content (Niknejad et al., 2008; Starecki et al., 2014; Lei et al., 2017; Witherel et al., 2017; Tang et al., 2018; Palanker et al., 2019; Sabouri et al., 2020).

This project explores the contribution of matrix *vs*. soluble biomolecules from the Amnion and Chorion membrane on mesenchymal stem cell osteogenesis and immunomodulatory potential. MSC are widely used in regenerative medicine due to their capacity to differentiate to a multitude of tissue-specific cell types, but are also increasingly considered for their immunomodulatory potential and capacity to modulate macrophage phenotype to enhance tissue repair (Eggenhofer and Dewey, 2012; Li and Hua, 2017; Zimmermann et al., 2017; Weiss and Dahlke, 2019; Wechsler et al., 2021). This project builds on a class of mineralized collagen scaffold previously reported by our group that promotes osteogenesis in the absence of osteogenic supplements or osteogenic media (Caliari and Harley, 2014; Weisgerber et al., 2015) and promotes mineral formation *in vitro* and *in vivo* (Ren et al., 2015; Weisgerber et al., 2018). These scaffolds do not inherently contain immune-regulatory components, motivating efforts to incorporate elements of the amnion membrane as an immunomodulatory modification, which was first investigated in non-mineralized collagen scaffolds for connective tissue repair (Hortensius et al., 2016; Hortensius and Harley, 2016). We subsequently showed amnion matrix added to the mineralized collagen scaffold improved MSC osteogenesis under inflammatory challenge (Dewey et al., 2020b). However, the specific contribution of placental derived membranes (soluble biomolecules, insoluble matrix) remains poorly understood. Here, we examine the osteogenic and immunomodulatory properties derived from amnion versus chorion membranes *via* inclusion of insoluble matrix or soluble biomolecule extracts from these membranes into the fabrication process used to create porous mineralized collagen scaffolds.

We report fabrication of five mineralized scaffold variants: conventional mineralized collagen scaffold (MC), mineralized collagen scaffold fabricated including amnion or chorion derived matrix particles (AMi or CMi), and mineralized collagen scaffold functionalized with amnion or chorion membrane derived soluble extracts (AMs or CMs). We hypothesized addition of the amnion and chorion membrane to mineralized collagen scaffolds would increase hMSC immunomodulatory properties while sustaining the osteogenic activity observed with our base mineralized collagen scaffold. Mineralized collagen scaffolds retained an open porous network after inclusion of amnion and chorion matrix with overall macroscopic porosity similar across all groups. Incorporation of amnion and chorion matrix significantly reduced scaffold elastic moduli, likely due to the inclusion of more organic content in the scaffold that may disrupt the scaffold mineral content which drives macroscopic mechanical performance. However, none of the scaffolds are designed to have macroscopic strength appropriate for direct bone implantation. The mechanics of low-density open-cell foams dictates that scaffold micromechanics required to support cell activity yields sub-optimal macroscale mechanical performance (Dewey et al., 2019). As a result, we have separately reported a reinforcement strategy that incorporates macroscale reinforcement mesh architectures into these scaffolds to support surgical practicality (Weisgerber et al., 2018; Dewey et al., 2021c). These meshes do not influence scaffold microarchitecture or negatively affect cell activity in scaffolds and were not included in this study as we focused on modification to scaffold composition.

We subsequently profiled the proteomic content of the amnion and chorion membrane, identifying a range of cytokines involved in bone formation and resorption, pro- and anti-inflammatory, and angiogenic functions. In agreement with Koob et al. (2015) and McQuilling et al. (2017) *,* we observed amnion and chorion contained similar amounts of cytokines when normalized per dry weight (Koob et al., 2015; McQuilling et al., 2017). The chorion extracts contained significantly higher levels of bone forming cytokines such as Leptin, IGFBP-1, and OPN all of which play significant roles in bone metabolism, osteoblast differentiation, and bone formation. The chorion extracts also contained significantly higher levels of immune cytokines such as HGF, and OSM known to induce inflammation and increase osteoclast expression, as well as angiogenic cytokines such as VEGF-A, PLGF, and RANTES. These upregulated cytokines would suggest that chorion containing scaffolds may more strongly influence osteoblast differentiation and bone formation but may also induce a pro-inflammatory response and osteoclastogenesis. We previously showed mineralized collagen scaffolds soaked in media containing soluble biomolecules efficiently sequestered these biomolecules within the scaffold microarchitecture (Tiffany et al., 2020), suggesting a route by which our scaffolds may incorporate soluble factors bound within the amnion or chorion matrix. The amnion extracts expressed significantly higher levels of OPG, a crucial inhibitor of osteoclastogenesis; notably, we previously showed that scaffolds which induce increased OPG production significantly improved craniofacial bone regeneration in a critical sized rabbit cranial bone defect model (Ren et al., 2019a; Ren et al., 2019b). Amnion extracts also contained significantly higher levels of IL8, a pro-inflammatory cytokine that amplifies neutrophil accumulation at inflammation sites in early stages of healing. This cytokine would suggest amnion containing scaffolds would have a greater anti-inflammatory influence, inhibiting osteoclastogenesis and allowing for bone formation. Go et al., 2017 reported both amnion and chorion extracts could promote osteogenic differentiation, but chorion extracts more substantially improved osteogenic differentiation (Go et al., 2017; Tang et al., 2018).

We subsequently examined the biologic activity of MSCs within mineralized collagen scaffolds containing amnion or chorion derived matrix (AMi, CMi) or soluble biomolecules (AMs, CMs). All scaffolds supported cell proliferation and metabolic health, though the highest levels were observed in an unmodified mineralized collagen scaffold control and scaffolds functionalized with amnion membrane derived soluble extracts. Alkaline phosphate activity, indicative of the presence of osteoblast cells and new bone formation, and quantification of calcium and phosphorous through ICP, indicating mineral formation, was less in the chorion incorporated variant at day 15 but consistent between scaffold groups by day 21. Notably, exogenous production and release of the osteoclast-inhibitory glycoprotein OPG (Ren et al., 2019b), as well as the osteogenic factor OPN (Sase et al., 2012; Kahles et al., 2014) suggested greater production in conventional mineralized collagen scaffolds and those containing amnion-derived matrix or functionalized with amnion-derived soluble extracts compared to the chorion variants. Taken together, this data suggest mineralized collagen scaffolds functionalized with amnion-derived soluble extracts (AMs) has potential to improve MSC viability without affecting mineral formation or osteogenesis.

We subsequently used transcriptomic analyses to more broadly profile MSC osteogenic and immunomodulatory activity in mineralized collagen scaffolds as a function of amnion and chorion modifications. MSCs in scaffolds containing amnion matrix increased expression of osteogenic genes such as BGLAP compared to the other scaffold groups. Chorion matrix incorporated scaffolds expressed lower levels of osteogenic genes such as RUNX2, COL1A2, and BMP2, a finding that, given the reduced Elastic modulus of scaffolds containing Chorion matrix, is consistent with our previous study showing increasing the microscale stiffness of mineralized collagen scaffolds induce a greater osteogenic response (Ren et al., 2015). Interestingly, scaffolds functionalized with amnion derived soluble extracts displayed a significant early-stage upregulation of immunomodulatory genes (*p* < 0.05) such as IL-6 and IL-8, which play significant roles in monocyte differentiation (Nakamura et al., 2004). In late stages of culture, MSCs in scaffolds containing amnion and chorion matrix displayed significant (*p* < 0.05) upregulation of CCL2, IL-6, IL-8, and HGF (*vs*. soluble extract functionalized groups) suggesting an important role for matrix associated stimuli in modulating immune response. MSCs secretion of CCL2 in response to inflammation *in vivo* is known to induce monocytes migration into the circulation (Shi et al., 2011). Finally, MSCs in scaffolds containing amnion matrix displayed significant (*p* < 0.05) upregulation of ANGPT1 (angiogenic gene) and expresses higher levels of VEGFA than the chorion matrix variant in late stages of culture. In sum, scaffolds containing amnion matrix most consistently maintained osteogenic expression while enhancing immunomodulatory, and angiogenic gene expression in MSCs compared to scaffolds containing only amnion derived soluble extracts or scaffolds containing chorion matrix or soluble extracts. Differences in osteogenic response may be associated with the decellularization process, as incomplete chorion membrane decellularization may result in the presence of cellular debris that can influence MSC activity.

Together, we report inclusion of amnion and chorion membrane-derived matrix or soluble extracts in a mineralized collagen scaffold influence MSC osteogenic, immunomodulatory, and angiogenic potential. A material that could promote osteogenesis and temporally modulate the inflammatory response may represent a promising method to improve regenerative potential. Notably, soaking the scaffolds in amnion soluble extracts displayed increased MSC metabolic activity compared to the chorion groups and retained osteogenesis, while incorporation of amnion matrix-maintained osteogenesis and enhanced immunomodulatory and osteogenic gene expression. These findings suggest future opportunity to explore potential combinatorial addition of both matrix-bound and soluble factor sequestered signals to further aid bone regeneration. However, the timeframes of availability of amnion or chorion derived soluble extracts added to *vs*. matrix incorporated into a biomaterial may offer future opportunities to alter the kinetics of wound healing and regenerative medicine. Further, MSC osteoprogenitors constitute only one element of many in the complicated landscape of bone repair that includes osteoclasts and immune cells such as macrophages. As a result, ongoing work is characterizing the influence of membrane particle and extract incorporation into mineralized collagen scaffolds on *in vivo* inflammatory response as well as on *in vitro* macrophage polarization*.* Future work will also examine the influence of amnion and chorion membrane additives on the crosstalk between osteoprogenitors and macrophage phenotypes to enhance the capacity of mineralized collagen scaffolds to temporally resolve inflammation and accelerate CMF bone regeneration in both *in vitro* and *in vivo* models. Lastly, recent discovery of matrix-resident extracellular vesicles embedded in tissues motivates future work to explore the impact of amnion or chorion derived vesicles on MSC cell behavior compared to released extracts (Huleihel et al., 2016). Here we defined specific methods for incorporating of amnion and chorion matrix into a biomaterial providing a reproducible pathway to identify mechanisms associated with key biological arbitrators of osteogenic and immunomodulatory responses. This approach will allow for future endeavors examining patient-to-patient placental membrane variability as a function of key factors such as parturition, gestational age, and post-processing methods.

## 5 Conclusion

We report the incorporation of amnion and chorion membrane matrix or soluble extracts into mineralized collagen scaffolds to enhance CMF bone regeneration. Isolation of membrane extracts from the matrix provided a lens to explore the relative contribution of matrix *vs*. biomolecule signals towards the immunomodulatory and pro-regenerative properties that have been attributed to placental derived membranes. Addition of amnion and chorion matrix particles decreased the compressive properties of the material while not influencing overall porosity. Soaking mineralized collagen scaffolds in soluble extracts isolated from amnion membrane induced the greatest increase of MSC viability without negatively influencing robust mineral formation previously observed in conventional mineralized collagen scaffolds. Addition of amnion membrane matrix to the mineralized collagen scaffold maintained MSC osteogenic, while it enhanced immunomodulatory and pro-angiogenic potential compared to other scaffold groups while inclusion of chorion membrane matrix in the scaffold architecture induced the lowest osteogenic response. Scaffolds containing amnion or chorion derived matrix showed the potential for immunomodulatory activity based on a larger transcriptomic screen. Further studies aim to examine the effects of both placental and cell donor variability based on sex. Overall, this study shows addition of amnion membrane derived matrix into a mineralized collagen scaffold or functionalization of the scaffold with amnion membrane derived soluble extracts may retain the osteogenic potential observed in conventional mineralized collagen scaffolds while providing a means of enhancing immunomodulatory and angiogenic activity in complex bone defects.

## Data Availability

The raw data required to reproduce these findings are available upon request to BH. The processed data required to reproduce these findings are available upon request to BH.
